# Fat-Dachsous Signaling Coordinates Cartilage Differentiation and Polarity during Craniofacial Development

**DOI:** 10.1371/journal.pgen.1004726

**Published:** 2014-10-23

**Authors:** Pierre Le Pabic, Carrie Ng, Thomas F. Schilling

**Affiliations:** Department of Developmental and Cell Biology, University of California, Irvine, Irvine, California, United States of America; University of Pennsylvania School of Medicine, United States of America

## Abstract

Organogenesis requires coordinated regulation of cellular differentiation and morphogenesis. Cartilage cells in the vertebrate skeleton form polarized stacks, which drive the elongation and shaping of skeletal primordia. Here we show that an atypical cadherin, Fat3, and its partner Dachsous-2 (Dchs2), control polarized cell-cell intercalation of cartilage precursors during craniofacial development. In zebrafish embryos deficient in Fat3 or Dchs2, chondrocytes fail to stack and misregulate expression of *sox9a*. Similar morphogenetic defects occur in *rerea/atr2a* ^−/−^ mutants, and Fat3 binds REREa, consistent with a model in which Fat3, Dchs2 and REREa interact to control polarized cell-cell intercalation and simultaneously control differentiation through Sox9. Chimaeric analyses support such a model, and reveal long-range influences of all three factors, consistent with the activation of a secondary signal that regulates polarized cell-cell intercalation. This coordinates the spatial and temporal morphogenesis of chondrocytes to shape skeletal primordia and defects in these processes underlie human skeletal malformations. Similar links between cell polarity and differentiation mechanisms are also likely to control organ formation in other contexts.

## Author Summary

Little is known about the mechanisms of cell-cell communication necessary to assemble skeletal elements of appropriate size and shape. In this study, we investigate the roles of genetic factors belonging to a developmental pathway that affects skeletal progenitor behavior: the atypical cadherins Fat3 and Dachsous2 (Dchs2), and REREa/Atr2a. We show that cartilage precursors fail to rearrange into linear stacks and at the same time misregulate expression of *sox9a*, a key regulator of cartilage differentiation, in zebrafish embryos deficient in Fat3 or its partner Dchs2. Similar cartilage defects are observed in *rerea* ^−/−^ mutants, and Fat3 interacts physically and genetically with REREa. Our results suggest that Fat3, Dchs2 and REREa interact to control polarized cell-cell intercalation and simultaneously control skeletal differentiation through Sox9. By transplanting cartilage precursors between wild-type and Fat3, Dchs2 or REREa deficient embryos we demonstrate that all three factors exert long-range influences on neighboring cells, most likely mediated by another polarizing signal. We propose a model in which this coordinates the polarity and differentiation of chondrocytes to shape skeletal primordia, and that defects in these processes underlie human skeletal malformations.

## Introduction

What are the mechanisms of cell-cell communication that mediate organ morphogenesis? Bones, for example, have many different sizes and shapes yet the individual and collective cell behaviors necessary to assemble these shapes remain largely unknown. During development, cartilage serves as the blueprint for much of the adult skeleton. Cartilage models of long bones, including the digits, are aligned into columns of discoid cells that resemble stacks of coins [1]. This basic arrangement is also found in other endochondral bones, including in the craniofacial skeleton where studies in zebrafish suggest that these stacks form by oriented cell intercalations [2]. Notably, cartilage differentiation and morphogenesis are initiated by *sox9*, but the morphogenetic pathway(s) activated by this transcription factor remain unknown [3], [4].

Cell-cell intercalations such as those that occur in cartilage are often regulated by planar cell polarity (PCP) pathways [5]. First described in Drosophila epithelia, PCP refers to coordinated polarity within a cell sheet [6]. More recently, vertebrates have been shown to utilize PCP factors not only in polarizing epithelia but also in orienting cell divisions and movements, such as the cell intercalations driving tissue convergence and extension during gastrulation, neurulation and kidney formation [5], [7]–[11]. Hallmarks of PCP in epithelia are the locations of polarized hairs or cilia protruding from cells. Similarly, primary cilia and their associated basal bodies/microtubule organizing centers (MTOCs) orient towards the leading edges of intercalating cells [12], and distally in chondrocytes in the digits of mouse limbs [13]. These coordinated polarity dynamics observed in migrating cells and chondrocytes suggest that PCP may also be part of the mechanism that controls skeletal morphogenesis.

Two main pathways regulate PCP independently in Drosophila: the Frizzled (Fz) pathway and the Fat/Dachsous (Dchs) pathway [14]. Both pathways are conserved in vertebrates and variously required for polarity of diverse tissues, including cochlear hair cells and hair follicles [5], [15]. In mice, components of the Fz pathway regulate oriented divisions and intercalations of chondrocytes in the growth plates of long bones [16], and Fat3/4 cooperate during fusion of vertebral arches [17]. However, little is known about requirements for the Fat/Dchs pathway in skeletal morphogenesis.

Four relatives of components of the Drosophila Ft/Ds pathway function in vertebrate PCP: 1) Ft, 2) Ds, 3) Four-jointed (Fj) and 4) Atrophin (Atro) [18]–[21]. In Drosophila, heterophilic binding of the protocadherins Ft and Ds mediates cell-cell adhesion and communication, while the Golgi kinase Fj phosphorylates their cadherin domains to modulate their binding affinity [22]–[24]. Atro also modulates signaling and binds the intracellular domain of Ft [20], but has a dual role as a transcriptional co-repressor that interacts with histone deacetylase (HDAC) [25], [26]. Ft or Ds mutant clones induced within imaginal discs trigger reversals of cell polarity outside the clone in one direction. In addition, Ds and Fj form opposing gradients across fly epithelia and interact with uniformly expressed Ft molecules [23], [27], [28]. This suggests a model in which the linear gradient of Ft/Ds heterodimers polarizes cell fields [29]. However, more recent quantitative analysis of these polarity reversals in the fly eye instead suggests that Ft and Ds interact to modulate a secondary signal that regulates long-range polarity [30]. Whether or not vertebrate Fat/Dchs signaling propagates polarity at a distance, utilizes molecular gradients, or interacts with other polarizing signals remains completely unknown in any tissue or organ.

Here we use the accessibility and miniature organization of the zebrafish jaw skeleton to investigate the genetic mechanisms of cartilage morphogenesis. We show that morphogenesis of polarized chondrocyte stacks results from oriented cell intercalations that depend upon Fat3, Dchs2 and REREa/Atr2a and their regulation of *sox9a* expression. Chimaeric analyses show that all three are required non-cell autonomously and over several cell-diameters for cartilage stacking and polarity, consistent with activation of a secondary signal that regulates polarized cell-cell intercalation. Fat3 and REREa interact physically and genetically, and our results suggest that Fat3 indirectly induces *sox9a* by preventing REREa from repressing it, while Dchs2 induces *sox9a* expression. *Sox9a* in turn activates *fat3* and *dchs2* expression. We propose a model in which Fat/Dchs signaling coordinates morphogenesis and differentiation of cartilage by the non-cell autonomous regulation of polarized cell-cell intercalation and *sox9a* expression.

## Results

### Cartilage stacking and polarity in the pharyngeal skeleton

**Figure 1:**
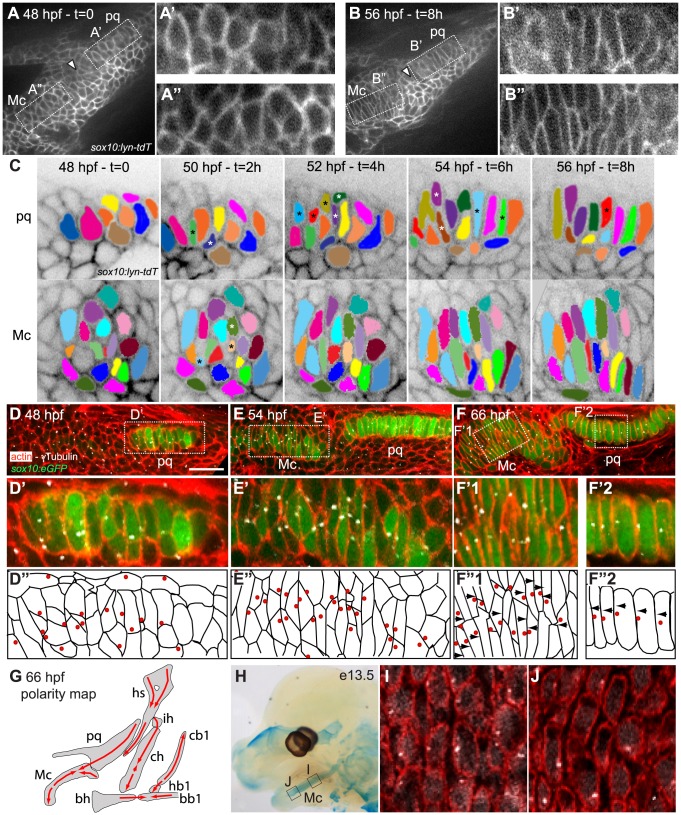
Morphogenesis and polarity of pharyngeal cartilages. (**A**–**B**): First (A) and last (B) time points of an 8 hour time-lapse movie of the first pharyngeal arch in a *sox10:lyn-tdTomato* transgenic, lateral view, anterior to the left. These frames show changes in cell shape and organization in presumptive palatoquadrate (pq) (A′ and B′) and Meckels (Mc) (A″ and B″) between 48 and 56 hpf. Arrowheads point to presumptive joint. (**C**). Color tracking of selected pq and Mc cells in the time lapse shown at 2 hour intervals. Asterisks denote medio-laterally intercalating cells. (**D**–**G**): Polarity dynamics during cartilage morphogenesis. Embryos stained for cortical actin with phalloidin (red) to reveal cell outlines, and anti-acetylated tubulin (white). (D) *sox10:eGFP* fluorescence first appears in differentiating chondrocytes of presumptive pq by 48 hpf, and in Mc by 54 hpf (E). (D′, D″, E′, E″) MTOCs of intercalating cells localize towards the center of the condensation. (F, F′_1_, F″_1_, F′_2_, F″_2_) Stable cell arrangement and polarity patterns are achieved by 66 hpf. (G) Polarity map of cartilages in pharyngeal arches 1–3 at 66 hpf, illustrated in lateral view, anterior to the left. (**H**–**J**) Polarity pattern in the e13.5 mouse Mc. (H) Alcian Blue stained e13.5 mouse head showing regions of Mc assayed for polarity. (I) proximal Mc is polarized ventrally, while distal Mc is polarized dorsally (J). Scale bar = 21µm. Mc: Meckel's; pq: palatoquadrate; PA: pharyngeal arch. Hm: hyosymplectic; ih: interhyal; ch: ceratohyal; bh: basihyal; cb1: ceratobranchial1; hb1: hypobranchial1; bb1: basibranchial.

**Figure 2:**
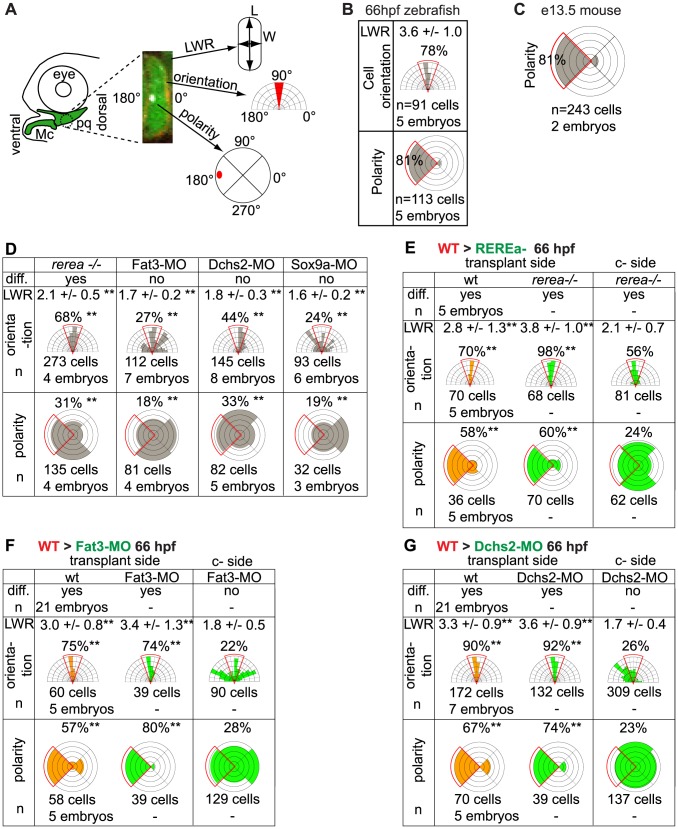
Quantification of differentiation, cell shape, cell orientation and cell polarity. (**A**) Cell length-width ratio (LWR) and orientation of longest cell axis were measured in pq to quantify cell stacking. Intracellular localization of microtubule organizing centers (MTOC) was recorded as readout of cell polarity. (**B**) LWR, cell orientation and polarity in WT zebrafish pq. (**C**) Polarity quantification in mouse proximal Mc. (**D**) LWR, cell orientation and polarity significantly differed (**) from WT values in all mutant and MO-treated embryos (p<0.001; Watson's U^2^ test). (**E**–**G**) LWR, cell orientation and polarity significantly differed (**) between transplant side and control side (p<0.001; Watson's U^2^ test). diff.: differentiation; c-side: control side. Mc: Meckel's; pq: palatoquadrate.

To understand the cellular basis of cartilage morphogenesis in the zebrafish pharyngeal skeleton we focused on pharyngeal arch 1 (PA1, mandibular), which in larvae consists of two elements, the ventral, lower – Meckel's cartilage (Mc) - and dorsal, upper – palatoquadrate (pq) - jaw cartilages. We conducted time-lapse analysis of pre-cartilage morphogenesis during the jaw-elongation period in a *sox10:lyn-tdTomato* transgenic driving membrane-localized red fluorescence in pharyngeal neural crest (NC) cells (Fig. 1A, B; Video S1) [31], [32]. Cell-cell rearrangements drive cartilage morphogenesis between 48-56 hpf. During this period, morphogenesis of the sheet-like pq (Fig. 1A′ B′) and rod-like Mc (Fig. 1A″, B″) was driven by a combination of radial and medio-lateral cell intercalations (Fig. 1C), while little cellular rearrangement occurred at the presumptive joint (arrowheads in Fig. 1A,B). Cell division did not contribute to growth of cartilage during this period but was observed in surrounding tissues (Video S1). EdU labeling confirmed the near absence of proliferation in intercalating prechondrocytes, as previously reported [2](Fig. S1A). Coupling of chondrocyte intercalation and differentiation was revealed in *sox10:eGFP* transgenics, where increased GFP fluorescence provides a readout of cartilage differentiation (Fig. 1D–F). A stable arrangement of chondrocytes in PA1 was achieved by 66 hpf. Quantification of chondrocyte morphology in pq revealed that in stacks the cell length to width ratio [LWR] is typically 3.6 +/− 1, with 78% of chondrocytes oriented perpendicular to the long axis of pq (n = 91 cells, 5 embryos) (Fig. 2A, B).

To characterize cell polarity during and after PA1 cartilage morphogenesis, *sox10:eGFP* transgenics were stained using an anti-gamma tubulin antibody to reveal the positions of microtubule organizing centers (MTOCs) [12], [33]–[35]. This revealed a dynamic pattern of polarity during the cell-cell intercalation period – 48-54 hpf, which stabilized by 66 hpf (Fig. 1D–F). Co-staining of acetylated alpha-tubulin showed that most MTOCs were associated with primary cilia (Fig. S1B). MTOCs in prechondrocytes within pq and Mc were initially oriented towards the center of each condensation (Fig. 1D″-E″). As Mc cell rearrangements stabilized by 66 hpf, three zones of uniform polarity became apparent along its dorsal-ventral (D–V) axis: 1) ventrally oriented near the jaw joint, 2) ventrally oriented near the midline joint with the contralateral Mc, and 3) dorsally oriented throughout the highly stacked region of Mc in between (Fig. 1F, G). In contrast, MTOCs were uniformly ventrally localized in pq at 66 hpf (Fig. 1F, G). Quantification of MTOC orientation in pq revealed 81% of chondrocytes were ventrally polarized (n = 113 cells, 5 embryos, Fig. 2A, B). Additional cryptic polarity reversals were observed throughout the cartilaginous skeleton of arches 2–7 at this stage (Fig. 1G).

To determine if such patterns of cartilage polarity are conserved across vertebrates, we stained MTOCs in Mc in mice. Similar to our data in zebrafish, chondrocytes in vibratome sections of Mc in mouse embryos at stages E12.5 and E13.5 were polarized (81% of MTOCs were ventrally oriented, n = 243, 2 embryos)(Fig. 1H, I and Fig. 2C). Furthermore, a distinct reversal in MTOC orientation was detected near the ventral (distal) end of Mc (Fig. 1H, J). Thus chondrocyte stacking, domains of coordinated polarity and cryptic boundaries between them are conserved in Mc in both fish and mammals.

### Fat3, Dchs2 and REREa are required for cartilage stacking and polarity

**Figure 3:**
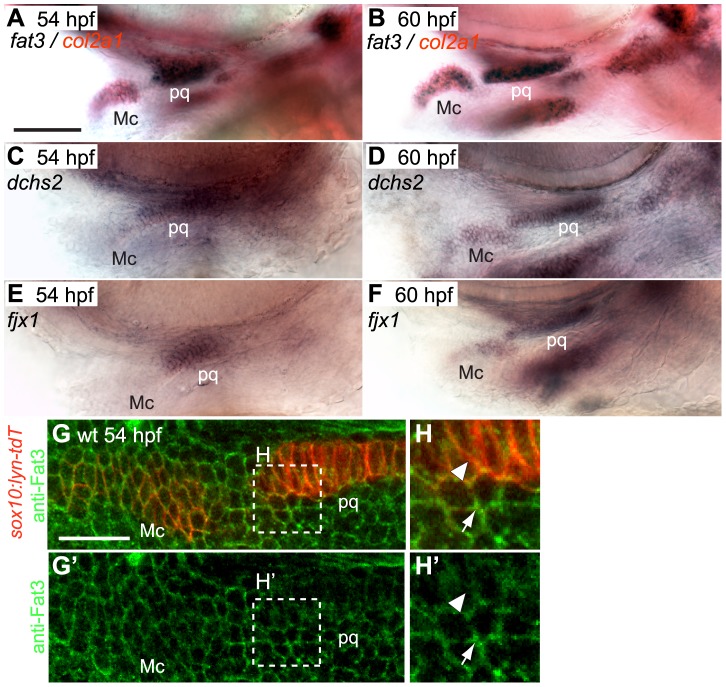
*fat3*, *dchs2* and *fjx1* are expressed in cartilage precursors. (**A**–**F**): In situ hybridization for *fat3*, *dchs2* and *fjx1*. Lateral views, anterior to the left. *fat3* (blue/black) is coexpressed with *col2a1* (red) in presumptive cartilages at 54 hpf (A) and 60 hpf (B). *dchs2* (C, D) and *fjx1* (E, F) are also expressed in presumptive pharyngeal cartilages at 54 and 60 hpf. Scale bar = 54 µm. (**G**–**H′**): Fat3 protein localizes to the cell membrane but is more diffuse in prechondrocytes than in surrounding cells. (G–H): Two-channel images showing *sox10:lyn-tdTomato* membrane labeling (red) and anti-Fat3 signal (green). (G′-H′): Single-channel images showing anti-Fat3 signal alone. Scale bar = 21µm. Mc: Meckel's; pq: palatoquadrate.

Cartilage stacking defects were previously reported in PA1 of zebrafish *atr2a/rerea^−/−^* mutants [36]. Drosophila Atro interacts with Ft in a common PCP pathway [20]. Therefore, we examined expression of multiple zebrafish Ft-, Ds- and Fj- orthologues – *rerea* is ubiquitously expressed in the head [36]. We found that *fat3*, *dachsous2 (dchs2)* and *four-jointed1 (fjx1)* are expressed in skeletogenic populations of all pharyngeal arches and the pectoral fin and co-expressed with *col2a1*, a marker for chondrocyte differentiation, between 48–72 hpf (Fig. 3A–F and Fig. S2A, B). *Fat3* expression is also detected at lower levels in cells surrounding skeletogenic areas (Fig. S2C, C′). Both *fat3* and *dchs2* are expressed in *rerea^−/−^* mutants (Fig. S2E, G). In order to visualize Fat3 protein localization, we generated an anti-Fat3 polyclonal antibody using the intracellular domain as epitope. Our antibody revealed that Fat3 localizes to the membranes of chondrogenic and non-chondrogenic pharyngeal NC cells at 54 hpf (Fig. 3G–H′), with stronger signal in non-chondrogenic areas (arrow).

**Figure 4:**
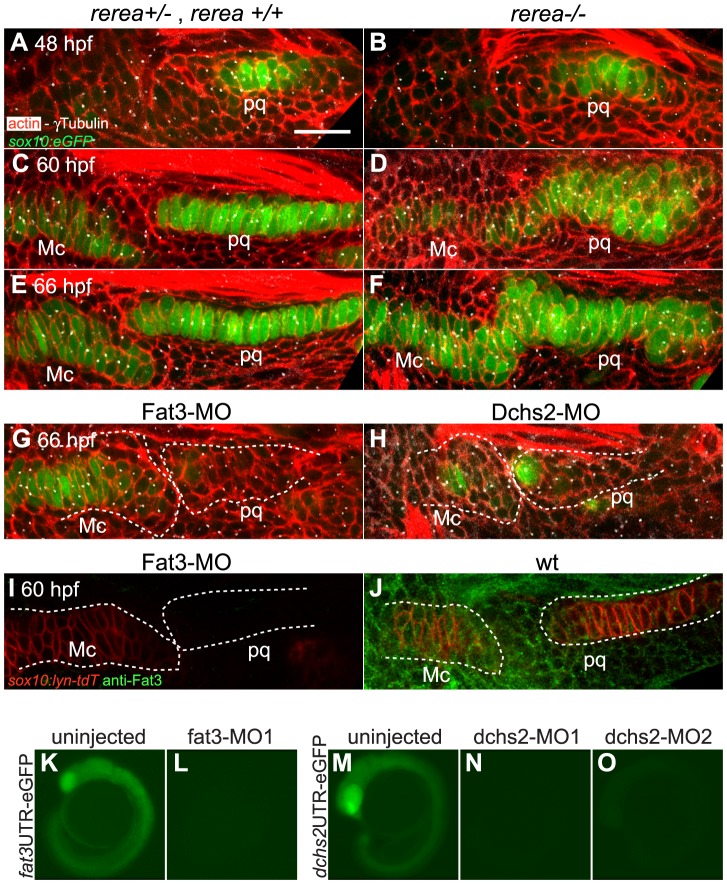
REREa, Fat3 and Dchs2 are required for cartilage stacking and polarity. (**A**–**F**): Abnormal stacking and polarity in *rerea^−/−^* embryos carrying the *sox10:eGFP* transgene (green) and stained for cortical actin with phalloidin (red) to reveal cell outlines. Lateral views, anterior to the left. (A, B) Stacking and polarity are comparable in *rerea^+/+^*, *rerea^+/−^* and *rerea^−/−^* pq at 48 hpf. (C, D) Stacking and polarity defects appear in *rerea^−/−^* pq by 60 hpf, and persist at 66 hpf (E–F). (**G, H**): Abnormal differentiation, stacking and polarity in Fat3- (G) and Dchs2-deficient embryos (H). (**I**–**J**): Fat3-MO injection decreases Fat3 protein levels (loss of green in I) in comparison to WT levels (J). Scale bar = 21µm. (**K**–**O**) eGFP reporter RNAs – *fat3* 5′UTR-eGFP (K,L) or *dchs2* 5′UTR-eGFP (M–O) – were injected alone or together with either fat3-MO1 (L), dchs2-MO1 (N) or dchs2-MO2 (O), and eGFP fluorescence measured at 18–20 hpf. Views are lateral, with anterior to the left. Mc: Meckel's; pq: palatoquadrate.

To test for potential roles for Fat/Dchs signaling in cartilage PCP, we investigated prechondrocyte intercalation and polarity in REREa-, Fat3- or Dchs2-deficient embryos in a *sox10:eGFP* background between 48–66 hpf. Stacking defects were observed in *rerea^−/−^* mutants as early as 60 hpf, suggesting a failure in cell-cell intercalation (Fig. 4A–F). Patterns of intercalating cell polarity, as assayed by MTOC position, in pq were generally similar in *rerea^−/−^* mutants to siblings at 48–60 hpf - MTOCs were oriented towards the center of the condensation. However, by 66 hpf, most chondrocytes in *rerea^−/−^* mutants failed to orient their MTOCs ventrally (Fig. 4F and Fig. 2D).

To test requirements for Fat3 in cartilage morphogenesis we designed two antisense morpholino oligonucleotides (MO) – fat3-MO1 and fat3-MO2, targeting the translation start site and a splice acceptor site, respectively. These MOs caused severe reductions in Fat3 protein levels, as assayed by whole mount immunostaining of PA1 in injected embryos (Fig. 4I, J), and fat3-MO1 suppressed eGFP expression when co-injected with *fat3*-5′UTR-eGFP mRNA (Fig. 4K, L). Both MOs caused similar cartilage differentiation defects at 66 hpf, with greater severity in dorsal elements, as revealed by loss of fluorescence in *sox10:eGFP* transgenics under epifluorescence microscopy (Fig. S3B; Table 1). A mixture of fat3-MO1 (23.1 nM) and fat3-MO2 (69.2 nM) gave consistent phenotypes when coinjected with p53-MO (7.7 nM) to eliminate non-specific apoptosis [37], [38] and was used in all subsequent experiments. To quantify defects in prechondrocyte shape and orientation we focused on pq at 66 hpf under confocal microscopy (Fig. 4G): Fat3-deficient cells were significantly less elongated, lost their perpendicular orientation, and coordinated polarity (Fig. 2D).

**Table 1:** Quantification of Fat3-MO and Dchs2-MO differentiation defects.

	fat3-MO1	fat3-MO2	fat3-MO1+2	dchs2-MO1	dchs2-MO1	dchs2-MO2	dchs2-MO2	dchs2-MO1+2
Amount (ng)	3	1	3+1	2	4	2	4	2+2
(n)	62	64	101	24	57	30	70	32
% with phenotype (n)								
Dorsal deletion	0 (0)	0 (0)	52 (52.5)	4.2 (1)	10.5 (6)	0 (0)	22.9 (16)	40.6 (13)
Dorsal reduction	45.2 (28)	2.3 (1.5)	38.1 (38.5)	25 (6)	64 (36.5)	0 (0)	41.4 (29)	25 (8)
Ventral deletion	0 (0)	0 (0)	24.3 (24.5)	8.3 (2)	1.8 (1)	0 (0)	55.7 (39)	75 (24)
Ventral reduction	38.7 (24)	9.4 (6)	31.2 (31.5)	20.8 (5)	98.2 (56)	0 (0)	31.4 (22)	15.6 (5)

Similarly, we designed two MOs against distinct regions of the Dchs2 translation start region, dchs2-MO1 and dchs2-MO2. Knock-down efficiency was demonstrated by coinjection of either MO with *dchs2*-5′UTR-eGFP mRNA, which suppressed eGFP expression (Fig. 4M–O). Both MOs (92.3 nM) caused differentiation defects of the pharyngeal skeleton at 66 hpf (Fig. S3C; Table 1). Similar to Fat3, Dchs2-deficient cells were less elongated and without coordinated orientation or polarity (Fig. 4H; Fig. 2D).

These results demonstrate that REREa, Fat3 and Dchs2 are required for cartilage stacking and polarity, consistent with a role in PCP. Fat3 and Dchs2 are also required for cartilage differentiation.

### Fat3, Dchs2 and REREa promote cartilage intercalation non-cell autonomously

**Figure 5:**
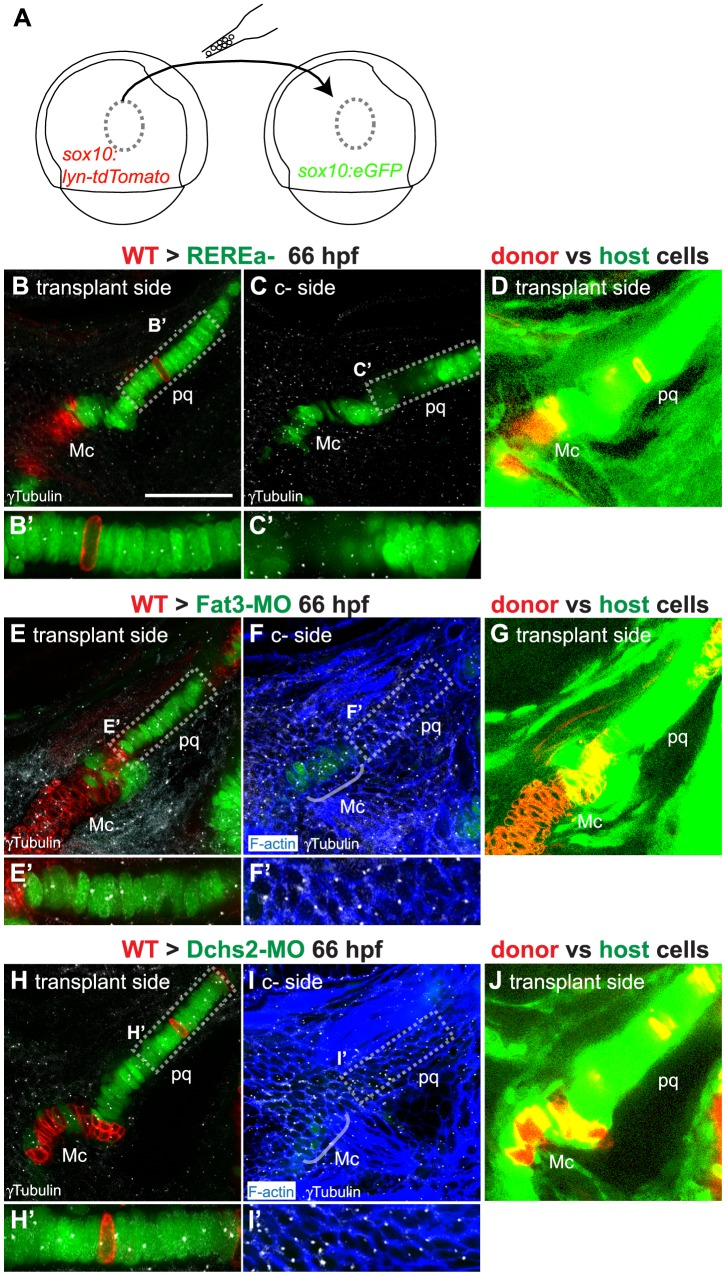
Non-cell autonomous requirements for REREa, Fat3 and Dchs2 in cartilage stacking and polarity. (**A**): presumptive CNC cells were transplanted from *sox10:lyn-tdTomato*- to *sox10:eGFP* transgenic embryos at the shield stage (**B**–**D**): WT transplants rescue cartilage stacking and polarity non-cell autonomously in *rerea^−/−^* embryos. Embryos stained for anti-acetylated tubulin (white). (B, B′) single confocal slice showing rescue of stacking and polarity in (*rerea^−/−^; sox10:eGFP*) mutant cells by a (WT; *sox10:lyn-tdTomato*) transplant. (C, C′) contralateral side without transplant. (D) Transplanted side with increased brightness in the green and red channels to show lineal contributions in non-cartilage cells. (**E**–**J**) Non cell-autonomous rescue of cartilage differentiation, stacking and polarity in (Fat3-MO; *sox10:eGFP*)(E, E′) or (Dchs2-MO; *sox10:eGFP*)(H, H′) embryos by (WT; *sox10:lyn-tdTomato*) transplants. (F, F′, I, I′) contralateral sides without transplants, stained for cortical actin with phalloidin (blue) to reveal cell outlines. (G, J) Transplanted side images with increased brightness in the green and red channels to show lineal contributions in non-cartilage cells. Scale bar = 50µm. Mc: Meckel's; pq: palatoquadrate.

A key aspect of PCP pathways is their role in coordinating the polarity/behavior of cell populations through cell-cell communication. We thus proceeded to test the non-cell autonomous requirements of REREa, Fat3 and Dchs2 in cartilage morphogenesis and differentiation by chimaeric analysis. WT NC cells from *sox10:lyn-tdTomato* transgenic donors were transplanted into *sox10:eGFP* hosts deficient in REREa, Fat3 or Dchs2 using the detailed fate map for the zebrafish gastrula (Fig. 5A). Chimaeras were screened for tdTomato+ NC cells at 24 hpf and raised to 66 hpf, when cartilage polarity and differentiation are spatially and temporally stabilized (see Fig. 1). The length-width ratio (LWR) and orientation of the long axes of cells were measured as assays of intercalation, while MTOC position was used to determine cell polarity.

WT transplants rescued intercalation in *rerea−/−* embryos (Fig. 2E; Fig. 5B–D). In chimaeras, both cartilage stacking and polarity of *rerea−/−* cells were rescued when compared with contralateral cartilages in the same animals, serving as an internal control. Notably, WT NC cells rescued stacking and polarity of REREa-deficient cartilage even when small numbers of transplanted WT cells contributed to the pq – 3 out of 5 chimaeras, exemplifying the long-range action of REREa in regulating cartilage morphogenesis.

WT NC transplants similarly rescued cartilage intercalation and polarity, but also differentiation, non-cell autonomously in both Fat3- and Dchs2-deficient embryos (Fig. 2F, G; Fig. 5E-J). WT transplants rescued stacking, polarity and differentiation - even when very small numbers of WT cells contributed to the pq - and long-range rescue was observed in 5 out of 21 Fat3-deficient embryos, and 6 out of 21 Dchs2-deficient embryos.

### Regulatory feedback between Fat3, Dchs2, REREa and Sox9a

**Figure 6:**
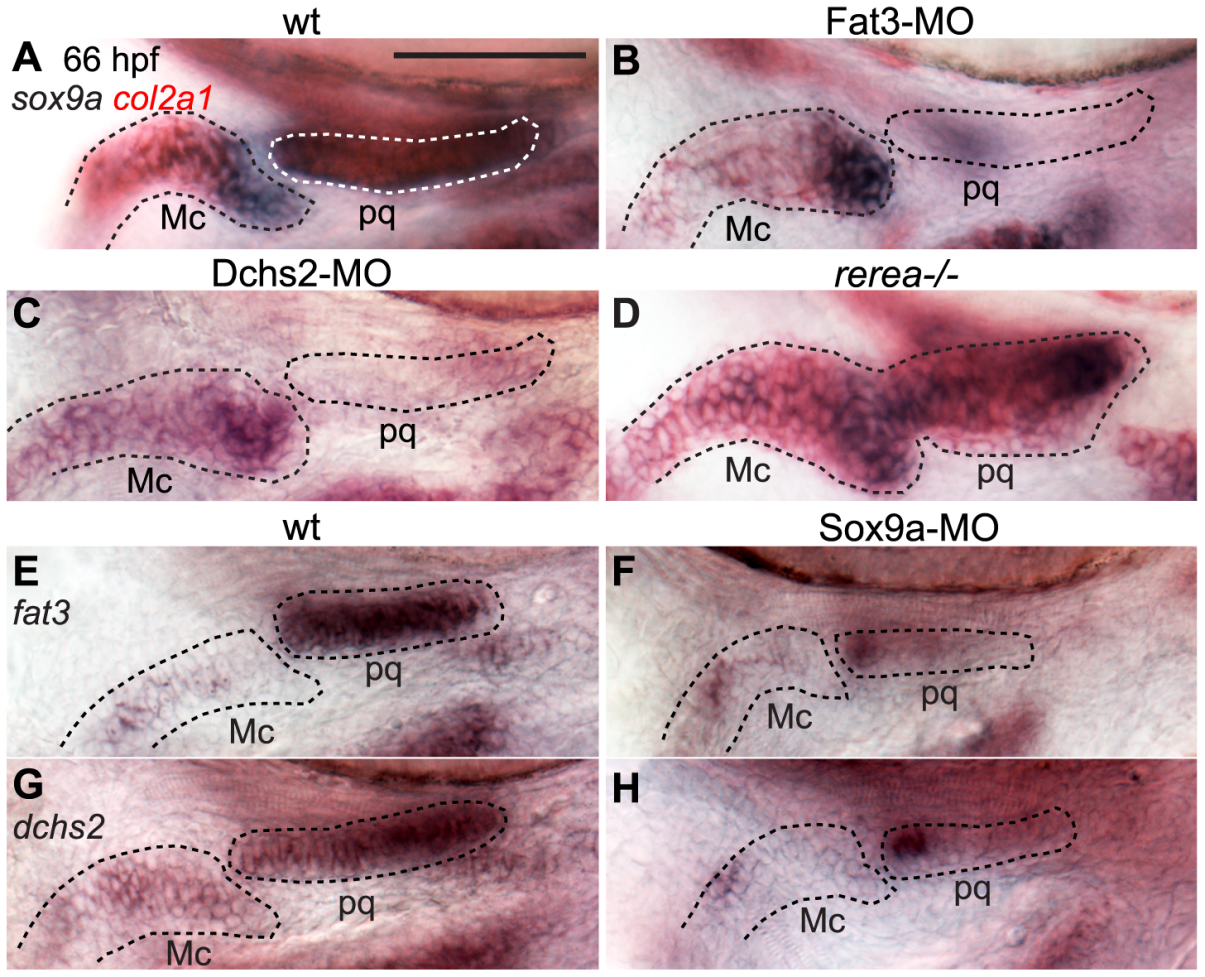
Regulatory feedback between Fat3, Dchs2, REREa and Sox9a. In situ hybridizations, lateral views, anterior to the left. (**A**–**C**) Reduction of *sox9a/col2a1* expression in Fat3- (B) or Dchs2-deficient embryos (C) at 66 hpf. (**D**) Ectopic expression of *sox9a/col2a1* in *rerea−/−* embryos at 66 hpf. (**E**–**H**) Reduction in Sox9a results in reduced *fat3* (F) and *dchs2* (H) expression.

*sox10:eGFP* fluorescence is strongly reduced in cartilage precursors of Fat3- or Dchs2-deficient embryos, indicating a differentiation defect. To examine this in more detail we assayed *sox9a* and *col2a1* expression by ISH. Expression of both genes was reduced or absent during the normal time-course of cranial cartilage differentiation (54–72 hpf) in both Fat3- and Dchs2-deficient embryos, with the dorsal arches affected at a greater frequency than ventral arches (Fig. 6A–C). In contrast, *sox9a* expression appeared spatially expanded in *rerea^−/−^* mutants, including ectopic expression in the presumptive jaw-joint interzone (Fig. 6D), as previously reported [36]. These results suggest that Fat3 and Dchs2 promote and REREa represses *sox9a* expression.

Previous studies have reported stacking and differentiation defects in *sox9a^−/−^* mutant embryos [4], which we confirmed by injecting a Sox9a-MO mix into *sox10:eGFP* embryos [4](Fig. S4). Sox9a-deficient embryos showed stacking and polarity defects comparable to Fat3- or Dchs2- deficient embryos (Fig. 2D). Analysis of *fat3* and *dchs2* mRNA by ISH revealed that both were reduced in the presumptive pharyngeal skeleton of Sox9a-, as well as Fat3- and Dchs2-deficient embryos (Fig. 6E–H, Fig. S5). These results show that Sox9a is required for the expression of *fat3* and *dchs2*, which in turn positively regulate *sox9a* expression.

### REREa interacts with Fat3 in a common pathway upstream of Sox9a

**Figure 7:**
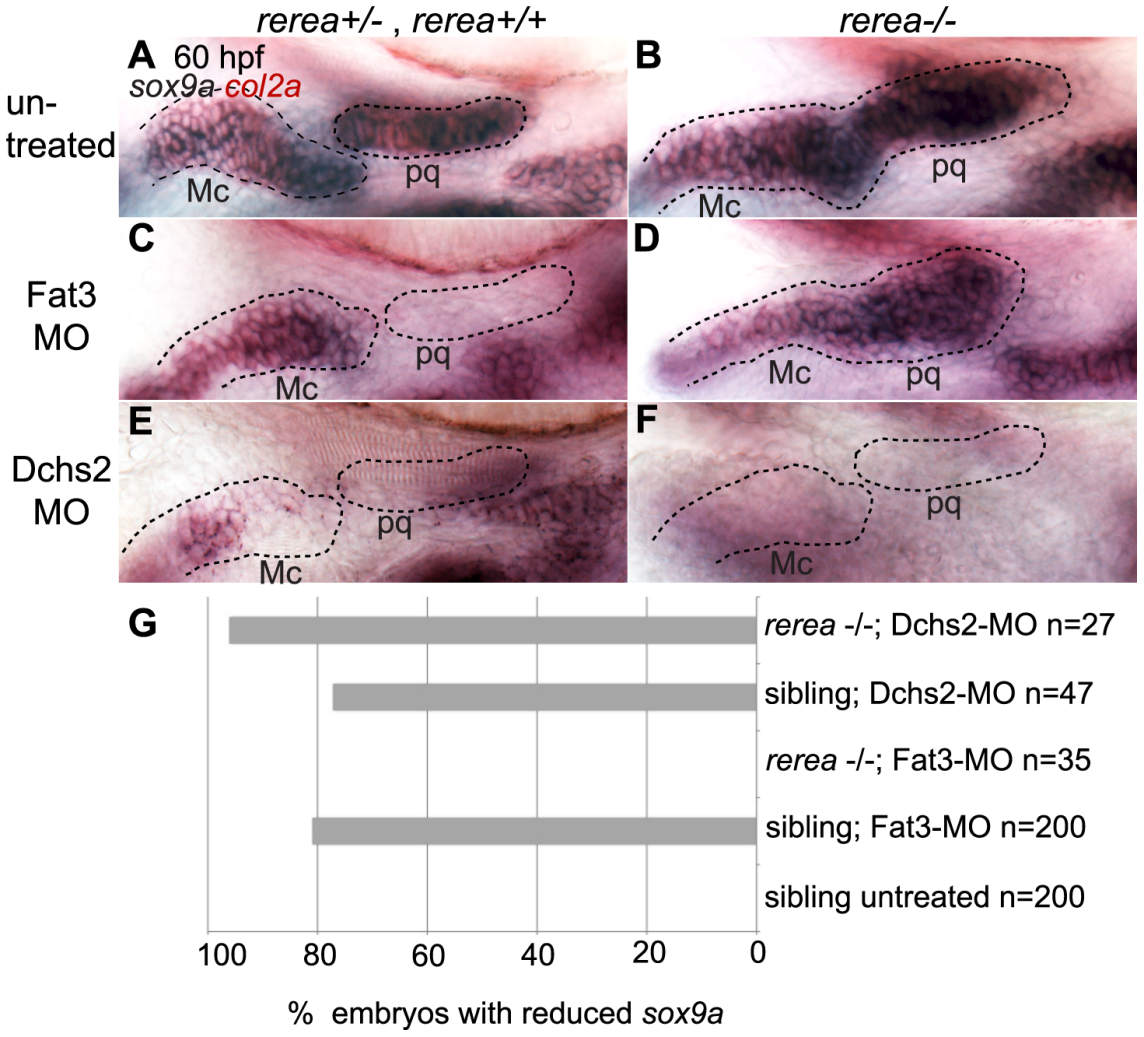
REREa interacts genetically with Fat3. (**A, B**) *sox9a/col2a1* expression patterns in *rerea^+/+^*, *rerea^+/−^* (A) and *rerea^−/−^* (B) embryos. (**C, D**) Reduction in *sox9a/col2a1* expression in Fat3-deficient embryos (C), but not in embryos deficient in both Fat3 and REREa (D). (**E, F**) Reduction in *sox9a/col2a1* expression in Dchs2-deficient embryos (E) and embryos deficient in Dchs2 and REREa (F). (**G**) Quantification of the proportion of embryos with reduced *sox9a/col2a1* expression. Scale bar = 54µm. Mc: Meckel's; pq: palatoquadrate.

To further investigate the functional relationship between Fat3, Dchs2 and REREa, we assayed *sox9a* and *col2a1* expression in embryos deficient in both REREa and Fat3, or REREa and Dchs2. *rerea^+/+^* and *^+/−^* embryos injected with Fat3-MO showed loss or reduction of *sox9a*/*col2a1* expression similar to WT embryos (n = 121)(Fig. 7C, G). In contrast, robust *sox9a/col2a1* expression was detected in the presumptive pharyngeal skeleton of all *rerea^−/−^* mutants deficient in Fat3 (n = 35) (Fig. 7D, G). This loss-of-function interaction suggests that REREa represses *sox9a* expression in Fat3-deficient tissues. *rerea^−/−^* embryos (n = 27) injected with Dchs2-MO showed reduced *sox9a/col2a1* expression, similar to Dchs2-MO injected siblings (n = 47)(Fig. 7E–G).

**Figure 8:**
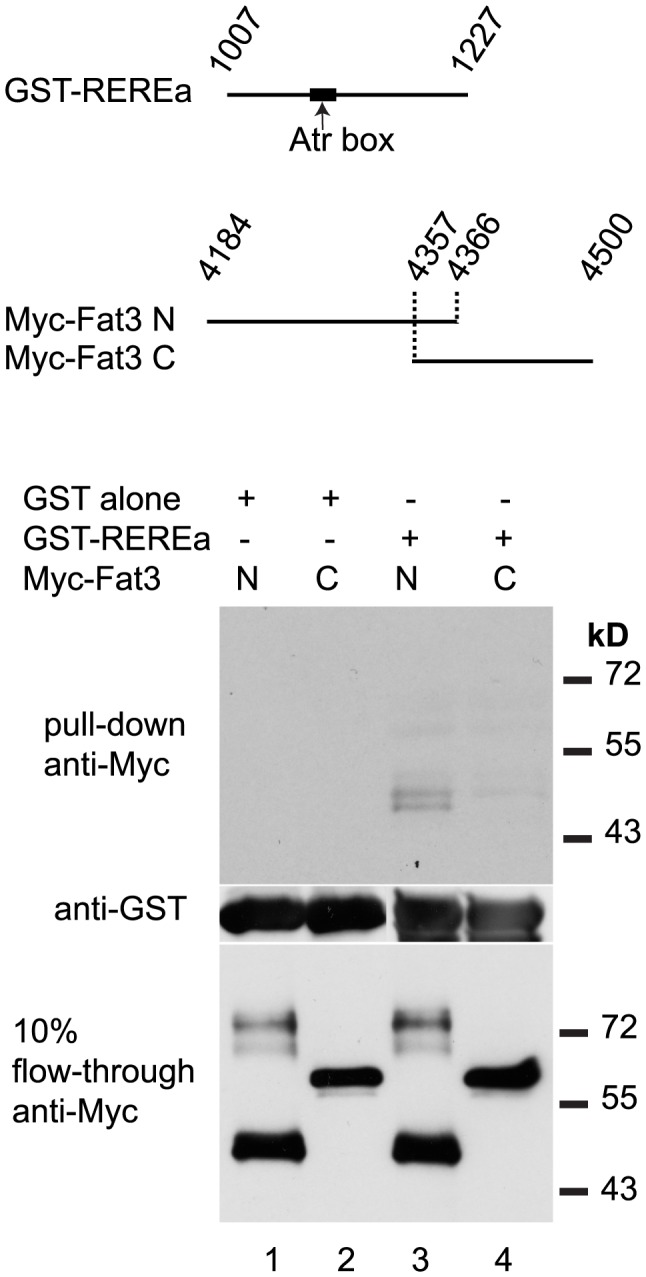
REREa and Fat3 bind each other in vitro. In vitro binding assay between GST-tagged fragment of the REREa atrophin domain and Myc-tagged N-terminal (N) or C-terminal (C) fragments of the Fat3 intracellular domain. Western blotting with anti-Myc antibody reveals the amount of Myc-Fat3 pulled-down by GST alone (columns 1–2) and GST-REREa (columns 3–4), respectively. Blotting with anti-GST antibody indicates the amounts of GST fusion proteins present. Blotting of 10% flow-through with anti-Myc indicates the amount of unbound Myc-Fat3 after pull-down.

Drosophila Atro binds Fat [20], as does Atr1 to Fat1 in mouse vascular smooth muscle cell primary cultures [39]. To test if zebrafish REREa/Atr2a binds Fat3 we performed in vitro binding assays with 2 non-overlapping fragments of the full-length Fat3 intracellular domain, together with a subset of the Atro domain of REREa containing the highly conserved Atr-box required for strong Fat-binding [39] (Fig. 8). We found that the N-terminal fragment of the Fat3 intracellular domain bound the Atro domain of REREa (Fig. 8). Our results are consistent with direct interactions between Fat3 and REREa upstream of *sox9a* expression. Taken together, these results suggest that Fat3 and REREa interact directly in a common pathway regulating *sox9a* expression. Further, the repressor activity of REREa on *sox9a* transcription appears to be inhibited by Fat3.

## Discussion

**Figure 9:**
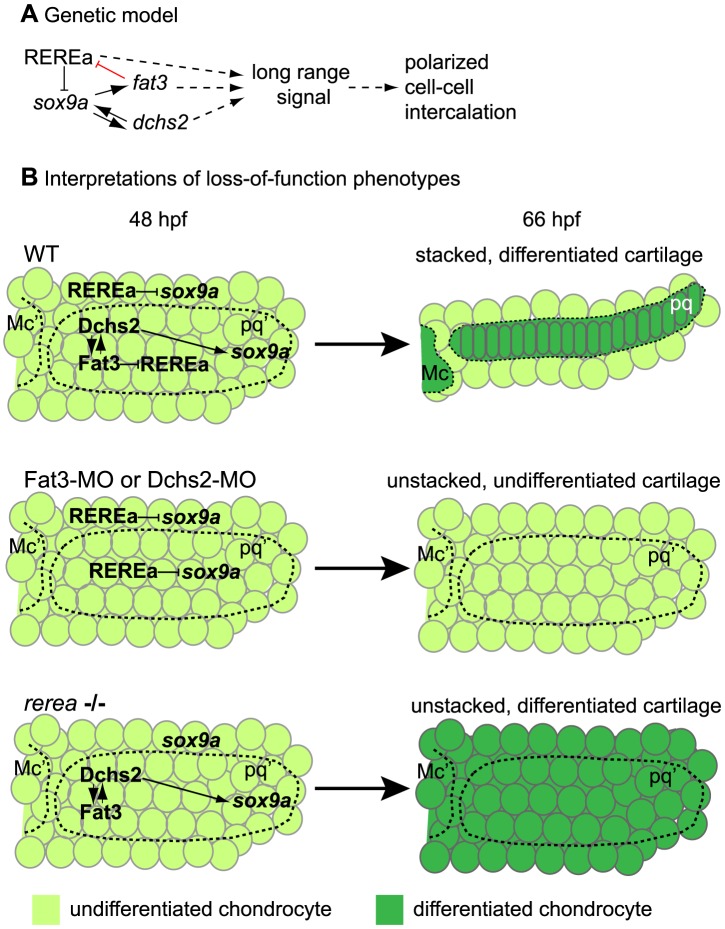
Summary and model. (**A**) Genetic interactions between REREa, Fat3, Dchs2 and Sox9a. Sox9a positively regulates *fat3* and *dchs2* expression. Fat3 acts indirectly as positive regulator of *sox9a* expression by preventing REREa repression (direct interaction in red), while Dchs2 positively regulates *sox9a* expression independently from REREa. Dotted lines indicate unresolved causal relationships within the REREa/Fat3/Dchs2/Sox9a cassette, (1) its long-range signaling potential and (2) its requirement in cell-cell intercalation. (**B**) Interpretation of Fat3/Dchs2/REREa loss-of-function phenotypes integrating the functional consequences of our genetic model on cartilage differentiation and morphogenesis in the zebrafish jaw. Mc: Meckel's; Mc': presumptive Meckel's; pq: palatoquadrate; pq': presumptive palatoquadrate.

In this study, we show that zebrafish pharyngeal cartilages – which form the blueprint for much of the adult craniofacial skeleton - are composed of polarized arrays of stacked chondrocytes. This neat organization is achieved through cell-cell intercalation and requires Fat3, Dchs2 and REREa (Fig. 9, Table 2). Our chimaeric analyses show that all three factors are required to promote polarized intercalation of prechondrocytes non-cell autonomously and over several cell diameters. While Fat3-Dchs2 bridges may rescue cells in contact with WT transplants in Fat3- or Dchs2-deficient embryos, the long-range rescue we observe likely reflects the activation of an unknown secondary signal that regulates long-range polarity. Furthermore, in our model Fat signaling plays more of a permissive role in cartilage morphogenesis and polarity, since we do not detect intercalation and/or polarity perturbations across boundaries between WT transplants and Fat3-, Dchs2- or REREa-deficient cells. Our results also provide a novel transcriptional link between Fat3/Dchs2/REREa and Sox9 in cartilage morphogenesis: REREa represses *sox9a* transcription (either directly or indirectly), which is antagonized by its interaction with Fat3, while Dchs2 activates *sox9a* expression. Sox9a is in turn required for *fat3/dchs2* expression.

**Table 2:** Summary of loss of function interactions.

Genotype/knockdown	Phenotype	Figure	Interpretation
*rerea^-/-^*	gain of *sox9a* expression	Fig. 6D	REREa represses *sox9a* expression
Fat3-MO	loss of *sox9a* expression	Fig. 6B, 7C	Fat3 promotes *sox9a* expression
Dchs2-MO	loss of *sox9a* expression	Fig. 6C, 7E	Dchs2 promotes *sox9a* expression
Sox9a-MO	loss of *fat3* and *dchs2* expression	Fig. 6F, H	Sox9a promotes *fat3* and *dchs2* expression
Fat3-MO/*rerea^-/-^*	rescue of *sox9a* expression	Fig. 7D	Fat3 antagonizes the repressor activity of REREa on *sox9a* expression
Dchs2-MO/*rerea^-/-^*	loss of *sox9a* expression	Fig. 7F	Dchs2 promotes *sox9a* expression

Whether or not vertebrate Fat/Dchs signaling controls a PCP mechanism analogous to fly epithelia continues to be debated, and whether or not it can do so in a mesenchymal tissue like the skeleton, remains unclear. However, *fat3*, *dchs2* and *fjx1* are all expressed in craniofacial skeletal precursors and both Fat3 and Dchs2 deficient cartilages lose coordinated polarity and fail to intercalate (similar to *rerea* ^−/−^ mutants), consistent with a PCP-based model. Further, Fat3, Dchs2 and REREa regulate polarized cell-cell intercalation at long-range, likely through the modulation of a secondary signal, as recently demonstrated for Drosophila Ft/Ds signaling [30]. Lastly, defects in oriented precartilage cell intercalations resemble defects in convergence and extension movements during gastrulation [8], [40], [41], which are considered a hallmark of impaired vertebrate PCP [5].

Sox9 is a well-known regulator of cartilage differentiation, but its roles in morphogenesis remain unclear. Human Sox9 heterozygous mutations cause Campomelic Dysplasia (CD), a condition characterized by several skeletal dysmorphologies including cleft palate and hypoplasia/bending of many endochondral bones [42]. While differentiation defects in CD can be explained by disruption of Col2a1 expression, a direct transcriptional target of Sox9 [43], dysmorphologies of precartilage condensations of Sox9 mutant humans, mice and zebrafish remain unexplained [4], [42]. Our results demonstrate that Sox9a is part of a regulatory loop consisting of Fat3, Dchs2, and the transcriptional co-repressor REREa, which we propose coordinates differentiation and morphogenesis. In our model, Sox9a regulates *fat3* and *dchs2* levels, which in turn control polarized cell-cell intercalations and also feedback through REREa to allow differentiation. This model is consistent with all of our chimaeric results and points to a novel role for Sox9a in cartilage polarity and stacking.

Our analysis of the intracellular localization of MTOC's/ciliary basal bodies reveals unexpected domains of cartilage polarity throughout the pharyngeal skeleton, both in fish and mice. It also provides a read-out of cell polarity – MTOCs organize the microtubule cytoskeleton, Golgi complex, and primary cilium [5] – that is governed by PCP signaling in gastrulating cells [9], [12] as well as cartilage cells of the digits in mouse limbs [13]. We find that MTOCs orient towards the core of skeletal condensations during cell intercalation, similar to MTOCs in cultured cells [44], and cells converging during gastrulation [12]. In later stages, MTOCs in cartilage relocate to either the dorsal or the ventral side of each stacked chondrocyte in both zebrafish and mice. Notably, distinct zones of ventrally- or dorsally-oriented MTOCs are juxtaposed within individual cartilage elements, and these zones do not correlate with any obvious anatomical boundaries such as joints. Do these switches in MTOC orientation reflect local sources of signaling molecules? Are they instructive for setting up domains of proliferation, local tissue interactions, or muscle-skeleton attachments?

*Fat3* is a vertebrate orthologue of Drosophila *fat2*/*fat-like* [45], which regulates PCP of actin fibers within the ovarian follicle epithelium, without demonstrated links to Ds or Atro [46]. In contrast, Drosophila *ft* – the orthologue of vertebrate *fat4*, regulates PCP in the eye, abdominal cuticle and wing discs, together with Ds, Fj, and Atro [18], [20], [27], [47]. We provide new genetic evidence for a requirement for Fat3, Dchs2 and REREa in a common pathway regulating morphogenesis and differentiation of vertebrate pharyngeal cartilage. A physical interaction between RERE/Atr2 and Fat1 was previously reported in murine smooth muscle cells [39], suggesting that the direct interaction between Fat and Atro orthologues may be generally conserved. Our study also suggests that some form of signal transduction occurs downstream of both Fat3 and Dchs2, with Fat3 modulating REREa activity and Dchs2 activating *sox9a* transcription. While a prevalent model proposes that Fat-Ds-mediated cell-cell communication is unidirectional, with Ds acting as ligand and Fat as receptor [29], our finding parallels that of recent studies suggesting signal transduction downstream of Ds in Drosophila [48]–[50].

The roles we have found for the Fat/Dchs pathway in cartilage cell intercalation and morphogenesis resemble those of the Fz-PCP pathway that have been described in various vertebrate contexts [5], [7]–[11], including the zebrafish pharyngeal skeleton [51], [52]. However, studies in Drosophila suggest that Fat-PCP and Fz-PCP pathways regulate tissue polarity independently [14]. What seems to set the Fat/Dchs pathway apart in cartilage, based on our findings, is its role in coordinating morphogenesis and differentiation through Sox9. Similarly, in the fly eye, Ft and Atro coordinate R3/R4 photoreceptor fate determination with polarity, suggesting a conserved role for this pathway in coupling polarity and differentiation [18], [20], [53]–[55]. These processes must be coordinated during the development of all tissues and organs, yet are rarely studied together and the underlying mechanisms remain unclear. Future studies are needed to clarify whether or not Fat signaling plays similar roles in other tissues (e.g. cranial neural tube, renal tubules, etc.) where it has been implicated [11], [17].

## Materials and Methods

### Ethics statement

All animals were handled in strict accordance with good animal practice as defined by the relevant national and/or local animal welfare bodies, and all animal work was approved by the University of California, Irvine Institutional Animal Care and Use Committee.

### Animals

Adult zebrafish of the *AB strain, carrying alleles of *rerea*/*atr2a^tb210^* (*bab*) [36], the transgenic reporter line *sox10:eGFP* [56], [57] or *sox10:lyn-tdTomato* [32] were maintained and staged as described [31], [58]. CD-1 and Rosa26^flox-mTRed-Stop-flox-mGFP^ mice were maintained as described [59]. Embryonic day 12.5 and 13.5 (E12.5 and E13.5) timed matings were setup by crossing CD1 females (Charles River) with Rosa26^flox-mTRed-Stop-flox-mGFP^males [60].

### Tissue labeling procedures

Whole-mount in situ hybridization was carried out as previously described (Thisse et al., 1993). *fat3, dchs2 and fjx1* amplicons were amplified from 54 hpf cDNA using the following primers: Fat3f: CTTCATCGCCTTCAGGAAGA, Fat3r: GGCGGGTAGTCAC TGTCAAT, Dchs2f: CCGAGGAAGAGACAGCAGAGG, Dchs2r: CGTATTCCTGGCTGGGCA AC, Fjx1f: GAGCAGCGGGTGTTCTGGAG, Fjx1r: CATCAATCCTGCTCTGCAATGTG. Each amplicon was subcloned into pCR4-TOPO (Invitrogen) following manufacturer's instructions, transformed into BL21 competent cells and sequenced. Published probes include *col2a1* [61] and *sox9a* [62].

Immunohistochemistry was performed with rabbit anti-gamma-Tubulin (1/100 Genetex GTX113286), mouse anti alpha-acetylated Tubulin (1/100 Sigma T6557) and Alexa Fluor 568 Phalloidin (1/50 Life technologies A12380). Fixed mouse heads were embedded in 5% agarose and 100 µm sections were cut on a vibratome.

Visualization of mouse embryonic cartilages was achieved by performing alcian blue staining as previously described [63]

### Microinjection of morpholino antisense oligonucleotides

Morpholino antisense oligos (MO) were designed to block translation or splicing (Gene Tools, Inc.) including: fat3-MO1, 5′-CCTTCACCTGTGCAAACAGAGAACA-3′; fat3-MO2, 5′-TGCCCTCTTGCTCAGTTCGGCTCAT-3′; dchs2-MO1, 5′-CATGTTCATGC-GAAAACATTAGCAG; dchs2-MO2, 5′-AGAAAGTCCGTGTGTAAAACTCCAT-3′; Sox9a i1d, 5′-AATGAATTACTCACCTCCAAAGTTT-3′ [62]; Sox9a i2d, 5′-CGAGTCAAGTTT-AGTGTCCCACCTG-3′ [62]. MOs were prepared at 1 mM in dH2O and stored at room temperature. To construct *fat3*-5′UTR-eGFP and *dchs2*-5′-UTR-eGFP reporter genes, cDNA amplicons containing target sites of MOs were subcloned in frame into the pCS2-eGFP vector. MO- and mRNA injection volumes were approximately 500 pL. A 4 hr developmental delay was usually observed with MO-injected- and *rerea^−/−^* embryos, which was corrected for throughout the study.

### Imaging

Embryos labeled by in situ hybridization were photographed on a Zeiss Axioplan 2 microscope, equipped with a MicroPublisher 5.0 RTV camera using Volocity software (Improvision). Fluorescent immunostained embryos were photographed on a Zeiss LSM780 confocal microscope using a 63x/1.15 W C-APO objective. For time-lapse imaging, embryos were imaged on a Nikon Eclipse Ti spinning disk microscope equipped with a 40x/1.15 WI Apo LWD objective. Approximately 100 um z-stacks were captured at 0.5 um intervals every 5 minutes for 8 hours. ImageJ/Fiji was used for image processing. Cell contours were hand-drawn in ImageJ and measured for length-width ratio (LWR) and orientation. Each cell was divided into 4 quadrants to determine MTOC position. Cell orientation and MTOC position were plotted as rosette diagrams and Watson's U^2^ tests for significance were conducted using Vector Rose (PAZ software). In Fat3-MO or Dchs2-MO embryos, LWR and orientation were recorded within a 3-to-4 cell-thick presumptive palatoquadrate (pq) region bordered posteriorly by the mandibular aorta, dorsally by the adductor mandibularis muscle (amm) and anteriorly by the presumptive jaw joint – at amm mid-length.

### Chimaeric analyses

WT, *sox10:lyn-tdTomato* donor embryos were injected with 3% rhodamine-dextran at the 1-2-cell stage and cells were transplanted into *sox10:eGFP* hosts at the shield stage (6 hpf). Host embryos with red fluorescent cells in the pharyngeal arches were sorted at 24 hpf and reared up to 66 hpf for immunochemistry. *rerea^−/−^* mutant hosts were identified by lack of pectoral fins and eye coloboma at 66 hpf.

### Biochemical analysis

For the GST pull-down assay, 2 *fat3* fragments were PCR-amplified and cloned in frame with the N-terminal Myc tag provided in the pCS2-MT vector. Myc-Fat3 N (aa 4184-4366) and C (aa 4357–4500) were then synthesized by in vitro translation using TNT Quick (Promega). GST-REREa (aa 1007–1227) was produced by PCR-amplification and cloned in frame into pGEX-4T-1 (Amersham) GST. GST-REREa protein was produced in BL21 cells, extracted in PBS with protease inhibitor cocktail (Sigma) and purified using glutathione-coupled beads (GE healthcare).

For binding assays, equal amounts of Myc-Fat3 were added to 100 µL of pre-equilibrated beads containing GST-fusions in HMK buffer [64] and rotated at room temperature for 3 hours. Beads were recovered, washed in HMK and analyzed by SDS-PAGE gels followed by western blotting with ECL chemoluminescence (Amersham). The following primary antibodies were used: mouse anti-Myc (a gift from J. Sosnik, 1∶5000), rabbit anti GST (GeneScript 1∶10,000).

## Supporting Information

Figure S1 Proliferation and polarity of the pharyngeal skeleton. (**A**) Reduced proliferation in stacking precartilage in *sox10:lyn-tdTomato* transgenic incubated in EdU for 1 hour at 48 hpf and assayed at 56 hpf. Lateral views, anterior to the left. (**B-B″**) Most pre-cartilage MTOCs, labeled with anti-acetylated tubulin (green) are associated with primary cilia, labeled with anti-gamma tubulin (blue). Scale bar = 21 µm.
(EPS)

Figure S2 *fat3* and *dchs2* expression in WT and *rerea^−/−^* embryos. In situ hybridizations, lateral views, anterior to the left. (**A**) *fat3* is expressed in presumptive cartilages of the posterior pharyngeal arches at 72 hpf, and expressed in the pectoral fin blade at 66 hpf (**B**). (**C, C′**) Lower levels of *fat3* expression are also detected in cells surrounding the chondrogenic area. (**D–G**) *fat3* (E) and *dchs2* (G) are expressed in presumptive cartilages of *rerea^−/−^* embryos. Scale bar = 54 µm. Mc: Meckel's; pq: palatoquadrate.
(EPS)

Figure S3 Differentiation defects in Fat3- and Dchs2-deficient embryos. (**A–C**) Loss of eGFP-fluorescence in (Fat3-MO; *sox10:eGFP*) (B) and (Dchs2-MO; *sox10:eGFP*)(C) deficient embryos at 66 hpf, as seen by epifluorescence microscopy, lateral views, anterior to the left. Scale bar = 54 µm.
(EPS)

Figure S4 Differentiation, stacking and polarity defects in Sox9a-deficient embryos. (**A**) untreated 66 hpf embryo. (**B**) Abnormal differentiation, stacking and polarity in Sox9a-deficient embryos carrying the *sox10:eGFP* transgene (green) and stained for cortical actin with phalloidin (red) to reveal cell outlines, and anti-acetylated tubulin (white). Lateral views, anterior to the left. Scale bar = 21 µm.
(EPS)

Figure S5 Reduced *fat3* and *dchs2* expression in Fat3- or Dchs2-deficient embryos. In situ hybridizations, lateral views, anterior to the left. *fat3* (**A**) and *dchs2* (**D**) expression in 60 hpf WT embryos. *fat3* and *dchs2* expression levels are reduced in Fat3- (**B, E**) or Dchs2- (**C, F**) deficient embryos. Scale bar = 54 µm.
(EPS)

Video S1 Time-lapse movie of skeletal morphogenesis in the first pharyngeal arch. *sox10:lyn-tdTomato* embryo photographed between 48 and 56 hpf at 1 frame/5 minutes. Lateral views, anterior to the left.
(AVI)
